# Synergistic Effects of Green Tea Extract and Ginger Supplementation on Endurance Performance and Thermal Perception in Normothermic and Cold Environments: A Randomized, Placebo-Controlled, Double-Blind Crossover Trial

**DOI:** 10.3390/nu17182949

**Published:** 2025-09-13

**Authors:** Abdullah Demirli, Süleyman Ulupınar, Merve Terzi, Serhat Özbay, Abdullah Bora Özkara, Cebrail Gençoğlu, Ibrahim Ouergui, Luca Paolo Ardigò

**Affiliations:** 1 Faculty of Sports Sciences, Istanbul University-Cerrahpaşa, 34320 Istanbul, Turkey; abdullah.demirli@iuc.edu.tr; 2 Faculty of Sports Sciences, Erzurum Technical University, 25050 Erzurum, Turkey; suleyman.ulupinar.erzurum@edu.tr (S.U.); serhat.ozbay@erzurum.edu.tr (S.Ö.); cebrail.gencoglu@erzurum.edu.tr (C.G.); 3 Faculty of Health Sciences, Istanbul Yeni Yüzyıl University, 34010 Istanbul, Turkey; merve.terzi@yeniyuzyil.edu.tr; 4 Faculty of Health Sciences, Karadeniz Technical University, 61080 Trabzon, Turkey; bora.ozkara@ktu.edu.tr; 5 High Institute of Sport and Physical Education of Kef, University of Jendouba, Kef 7100, Tunisia; 6 Research Unit: Sport Sciences, Health and Movement, UR22JS01, University of Jendouba, Kef 7100, Tunisia; 7 Department of Teacher Education, NLA University College, 0166 Oslo, Norway

**Keywords:** supplementation, fat oxidation, thermal sensation, muscle soreness, cold environment

## Abstract

**Background/Objectives**: This study assessed the individual and combined effects of green tea extract and ginger supplementation on endurance performance, metabolic responses, perceived exertion, thermal sensation, and muscle soreness in normothermic and cold environmental conditions. **Methods**: In a randomized, double-blind crossover trial, sixteen recreationally active males (age: 23.4 ± 0.4 years; VO_2_ max: 46.8 ± 2.8 mL/kg/min) were tested in eight conditions (placebo [maltodextrin], green tea [500 mg], ginger [1000 mg], combined), all in normothermic (21–24 °C) and cold (5–7 °C) environments. All supplements and the placebo were encapsulated in identical capsules to ensure blinding. Participants completed a submaximal time-to-exhaustion (TTE) test at 70% VO_2_ max on a cycle ergometer. TTE, respiratory exchange ratio (RER), perceived exertion (RPE), thermal sensation (TSS), and muscle soreness via a visual analog scale (VAS), assessed 24 h post-exercise, were measured. **Results**: In normothermic condition, green tea and combined supplementation significantly increased TTE and reduced RER compared to the placebo (*p* < 0.05), and that combined supplementation lowered RPE relative to the placebo and ginger (all *p* < 0.05). In cold conditions, combined supplementation significantly enhanced TTE, reduced RER, and improved TSS compared to the placebo and ginger (*p* < 0.05), while all supplements decreased VAS relative to the placebo (*p* < 0.05). Ginger alone showed no significant effect on TTE or RER but improved TSS and VAS in cold compared to the placebo (*p* < 0.05). Cold placebo conditions exhibited significantly higher RPE and VAS than all normothermic conditions (*p* < 0.05). **Conclusions**: Green tea enhances endurance and fat oxidation in normothermic conditions, while its combination with ginger can optimize performance, thermal comfort, and recovery in cold environments. These findings suggest a practical nutritional strategy for mitigating environmental stress during exercise, specific to the acute supplementation in males. Trial Registration: This trial was registered at ClinicalTrials.gov (Identifier: NCT07150533).

## 1. Introduction

It is well known that endurance performance represents a cornerstone of exercise physiology, deeply influenced by environmental conditions and nutritional interventions [1,2]. Cold environments naturally impose significant physiological stress by increasing metabolic demands through thermogenesis and altering substrate utilization, often favoring carbohydrate over fat metabolism [3,4]. Such conditions can alter exercise capacity by increasing perceived exertion and diminishing thermal comfort, thereby accelerating the onset of fatigue during prolonged physical activity [5,6]. As athletes and recreational exercisers increasingly train and compete under diverse environmental stressors, the use of ergogenic aids in optimizing performance and mitigating environmental impacts has become of growing scientific interest worldwide [7,8].

Cold environments elicit distinct physiological responses that alter endurance performance and thermal homeostasis, particularly at temperatures of <10 °C [4,9]. Such conditions trigger non-shivering thermogenesis, elevating oxygen consumption (VO_2_) and shifting substrate metabolism toward increased fat oxidation, as indicated by a reduced respiratory exchange ratio (RER) [10,11]. While this metabolic adaptation may enhance energy efficiency, it is often accompanied by an increase in both thermal discomfort and perceived exertion, which can result in early fatigue and impaired exercise performance [6,12].

In this consideration, green tea extract and ginger have emerged as a promising ergogenic aid in the sports nutrition field, thanks to their bioactive constituents with well-documented physiological effects [13,14,15]. Green tea, enriched with catechins such as epigallocatechin gallate and caffeine, enhances fat oxidation and increases energy expenditure, which may contribute to modest improvements in endurance performance during submaximal exercise [16,17]. This is not surprising since caffeine is known for its central nervous system stimulant effect, potentially reducing perceived exertion, while catechins facilitate lipolysis, thereby conserving glycogen reserves that are indispensable for exercising during prolonged activity [18,19].

Likewise, ginger exhibits anti-inflammatory properties and mild thermogenic activity, due to its gingerols’ content, reducing muscle soreness, and enhances thermal comfort through improved peripheral circulation [8,15,20]. These individual mechanisms suggest that green tea and ginger may independently support endurance and thermoregulatory responses, particularly under cold stress, where enhanced thermogenesis and improved circulation may help counteract discomfort and fatigue, providing a scientific framework to raise the question of how they might interact synergistically.

Given the thermogenic properties of green tea and ginger that were previously underlined, these supplements may counteract the adverse effects of cold exposure by enhancing heat production and improving thermal perception, while their ergogenic effects could delay the onset of fatigue [8,9,16]. However, the potential for a synergistic interaction between green tea and ginger in cold environments remains largely unexplored, despite their complementary mechanisms, highlighting a critical gap in understanding how these supplements can optimize performance under such conditions. To our knowledge, no previous study has systematically examined the combined effects of green tea and ginger supplementation under both normothermic and cold environments within the same randomized, double-blind, crossover design. This positions the present study as the first to provide direct evidence of their potential synergistic efficacy in mitigating cold-induced decrements in endurance performance and thermal comfort. Therefore, this study aimed to evaluate the individual and combined effects of green tea extract and ginger supplementation on endurance performance, metabolic responses, and thermal perception in normothermic (21–24 °C) and cold (5–7 °C) environments using a randomized crossover design. We hypothesized that both supplements would individually improve endurance and thermal perception compared to the placebo, with combined supplementation eliciting greater improvements, particularly in the cold.

## 2. Materials and Methods

### 2.1. Study Design

This study employed a randomized, placebo-controlled, double-blind, crossover design to investigate the combined effects of green tea extract and ginger supplementation on endurance performance and thermal perception in normothermic (21–24 °C) and cold (5–7 °C) environmental conditions. The crossover design ensured that each participant served as their own control, thereby minimizing inter-individual variability. Furthermore, repeated measures within participants, combined with randomisation and washout periods, helped to reduce the influence of intra-individual variability across testing sessions. Eight experimental conditions were tested: placebo, green tea extract (500 mg per capsule; Ocean^®^, Orzaks Pharmaceutical and Chemical Industry Inc., Istanbul, Turkey) standardized to ~45% epigallocatechin gallate (EGCG; ≈225 mg per capsule), ginger extract (1000 mg per capsule; VeNatura^®^, Istanbul, Turkey), and a combined supplement, each administered in both normothermic and cold environments. All active and placebo supplements were provided in identical capsule form, prepared to be indistinguishable in appearance and packaging. The selected doses are consistent with previous clinical and sports nutrition studies that demonstrated efficacy and safety at these levels [10,11,15]. Green tea extract and ginger were administered orally in encapsulated form, identical in appearance to the placebo capsules filled with maltodextrin.

Participants were divided into groups of two, and conditions were administered in randomized orders using a computer-generated sequence (RandList software, version 1.2). A Latin square design was used to counterbalance order effects, ensuring each condition appeared in each position an equal number of times. Allocation was concealed using sequentially numbered, opaque envelopes to prevent selection bias. A 2 day washout period separated each condition to eliminate active compounds (e.g., caffeine half-life ~5 h, gingerols metabolized within 24 h), supported by prior pharmacokinetic studies [12,21,22,23]. The study was conducted in a temperature-controlled laboratory chamber (normothermic: 21–24 °C, cold: 5–7 °C; 40–50% humidity). Double-blinding was achieved by matching the appearance, taste, and packaging of the placebo (maltodextrin) and active supplements, prepared by an independent pharmacist unaware of the study protocol. To address potential circadian variability or acclimatization, all testing sessions were scheduled between 16:00 and 18:00 to control for diurnal effects.

### 2.2. Participants

Sixteen healthy male participants were recruited for this study, (Mean ± SD; age: 23.4 ± 0.4 years, height: 176.9 ± 3.5 cm, body mass: 74.1 ± 4.4 kg, body mass index (BMI) of 23.7 ± 0.7 kg/m^2^, body fat percentage: 14.6 ± 2.3%, and maximum oxygen consumption (VO_2_ max): 46.8 ± 2.8 mL/kg/min). Participants were classified as Tier 2: Trained/Developmental Active according to the Participant Classification Framework [24], as they met World Health Organization physical activity guidelines (≥75 min/week vigorous-intensity activity) but did not identify with a specific sport or compete. Inclusion criteria required participants to be aged 18–35 years, engage in regular aerobic exercise (≥3 sessions/week, ≥30 min/session) for at least 6 months prior to the study, and exhibit a VO_2_ max between 40 and 50 mL/kg/min, ensuring a homogeneous fitness level. Exclusion criteria included a history of cardiovascular, respiratory, or metabolic disorders, allergy or intolerance to green tea, ginger, or maltodextrin, current use of ergogenic supplements or medications affecting metabolism (e.g., beta-blockers and stimulants), and smoking or excessive alcohol consumption (>14 units/week). To address potential gender-related variability, only male participants were included to control for hormonal influences on thermoregulation and metabolism, a decision informed by prior research on sex differences in cold tolerance. All participants provided written informed consent prior to participation, in accordance with the Declaration of Helsinki. The study protocol was approved by the Yeni Yüzyıl University Ethics Committee for Science and Health Sciences Not Requiring Medical Intervention (Date: 4 March 2025; Protocol No: 2025/03-1509) and was registered at ClinicalTrials.gov (Identifier: NCT07150533).

### 2.3. Procedures

Prior to experimental trials, a familiarization session was conducted 48–72 h before the first trial to reduce learning effects. During this session, participants performed a VO_2_ max test on a cycle ergometer (Lode Excalibur Sport, Lode BV, Groningen, The Netherlands), calibrated before each use. The VO_2_ max test followed a graded exercise protocol starting at 50 W, increasing by 25 W every 2 min until volitional exhaustion or inability to maintain a 60 rpm cadence [25,26]. VO_2_ max was defined as the highest 30 s average oxygen consumption (Cosmed K5 portable gas analyzer, COSMED, Rome, Italy) with a respiratory exchange ratio ≥ 1.20, a VO_2_ plateau, or maximal heart rate within 10 bpm of the age-predicted maximum (220−age). VO_2_ max testing was conducted under normothermic conditions to ensure methodological consistency and comparability across participants, as cold exposure may introduce additional variability and confound the determination of relative exercise intensity.

Each experimental session began with a 10 min acclimatization period in a seated position to stabilize physiological responses under standardized conditions: normothermic (21–24 °C, 40–50% relative humidity) or cold (5–7 °C, 40–50% relative humidity). Participants performed a submaximal time-to-exhaustion test (TTE) at 70% of their individual VO_2_ max on the cycle ergometer. This intensity was chosen because ~70% VO_2_ max is a standard workload in endurance research, high enough to elicit substantial physiological stress while allowing prolonged exercise duration, thereby providing sensitivity to detect supplementation effects [27]. Exhaustion was defined as the inability to maintain a 60 rpm cadence for 10 consecutive seconds, measured via the ergometer’s software (Lode Ergometry Manager, version 10.5). TTE (min) was the primary measure of endurance performance. Metabolic responses were monitored continuously using the gas analyzer and calibrated per manufacturer guidelines (oxygen sensor accuracy: ±0.02%, carbon dioxide (CO_2_) sensor accuracy: ±0.01%). RER was calculated as the VCO_2_/VO_2_ ratio, averaged over the test duration for steady-state conditions. Perceived exertion was assessed using the Borg RPE scale (6–20) immediately post-exhaustion [28]. Thermal sensation (TSS) was measured pre-exercise (post-acclimatization) and post-exercise using a 7 point scale [−3: very cold to +3: very warm, 0.5 increments] [29]. Delayed onset muscle soreness (DOMS) was evaluated 24 h post-exercise via a 10 cm Visual Analog Scale [VAS; 0: no pain, 10: extreme pain], with participants instructed to report muscle soreness in the exercised lower limbs [30].

To standardize conditions, participants refrained from caffeine, polyphenol-rich foods (e.g., tea, coffee, cocoa, grapes, red wine, berries, and dark chocolate), dietary supplements, alcohol, and strenuous exercise for 48 h prior to each session. They also wore standardized clothing (shorts, t-shirt, athletic shoes; estimated insulation value ≈0.3–0.4 clothing insulation unit, CLO [31,32], and consumed 500 mL of water 2 h before testing to ensure hydration. Testing occurred between 16:00 and 18:00 to control for circadian effects, with participants maintaining a consistent sleep schedule (7–9 h/night). Dietary intake was controlled 24 h prior to each session, providing ~55% carbohydrates, 30% fats, and 15% proteins based on individual energy needs [33,34,35]. Food logs were reviewed for compliance.

### 2.4. Statistical Analysis

A priori power analysis was conducted using G*Power (version 3.1.9.4) to determine the required sample size for detecting significant effects in a two-way repeated-measures analysis of variance (ANOVA) (within factors). The analysis assumed an effect size (f) of 0.25 (equivalent to a partial eta squared, ηp^2^, of 0.06, indicating a medium effect), a significance level (α) of 0.05, and a desired power (1 − β) of 0.80. Additional parameters included a correlation among repeated measures of 0.5, a non-sphericity correction (ε) of 1, 4 groups (supplementation conditions: placebo, green tea, ginger, or combined), and 2 measurements (environmental conditions: normothermic or cold). The analysis yielded a required sample size of 16 participants to achieve a total power of 0.820, with a critical F-value of 2.098 and a non-centrality parameter (λ) of 16.0. Statistical analyses were performed using SPSS software (version 27.0, IBM Corp., Armonk, NY, USA). Data are presented as means ± standard deviations (SD). Data sets were first assessed for normality using the Shapiro–Wilk test and for sphericity using Mauchly’s test, ensuring the assumptions for parametric testing were met. In the case of sphericity violations, a Greenhouse-Geisser correction was applied to adjust degrees of freedom. A two-way ANOVA with repeated measures was used to examine the main effects of supplementation (placebo, green tea, ginger, combined) and environmental condition (normothermic, cold), as well as their interaction on each dependent variable. Partial eta squared (ηp^2^) was calculated to estimate effect sizes, with values interpreted as small (0.01), medium (0.06), or large (0.14) effects [36]. When significant main effects or interactions were identified, post hoc comparisons were performed using Bonferroni correction to control for Type I errors across multiple comparisons. To address potential concerns of over-conservativeness, results were also checked using Holm correction [37]. The most borderline pairwise comparison observed (*p* = 0.08) did not reach statistical significance under Holm adjustment, and the overall pattern of findings remained unchanged. The significance level was set at α = 0.05. For pairwise comparisons, Hedge’s g effect sizes were also computed and interpreted according to Hopkins’ criteria (trivial: <0.2, small: 0.2–0.6, moderate: 0.6–1.2, large: 1.2–2.0, very large: 2.0–4.0, and nearly perfect: >4.0) to provide additional insights into the magnitude of differences between conditions [38]. Hedges’ g was preferred over Cohen’s d due to its correction for small sample size bias, providing a more accurate estimate of effect size in repeated measures designs with modest participant numbers [39,40].

## 3. Results

**Figure 1:**
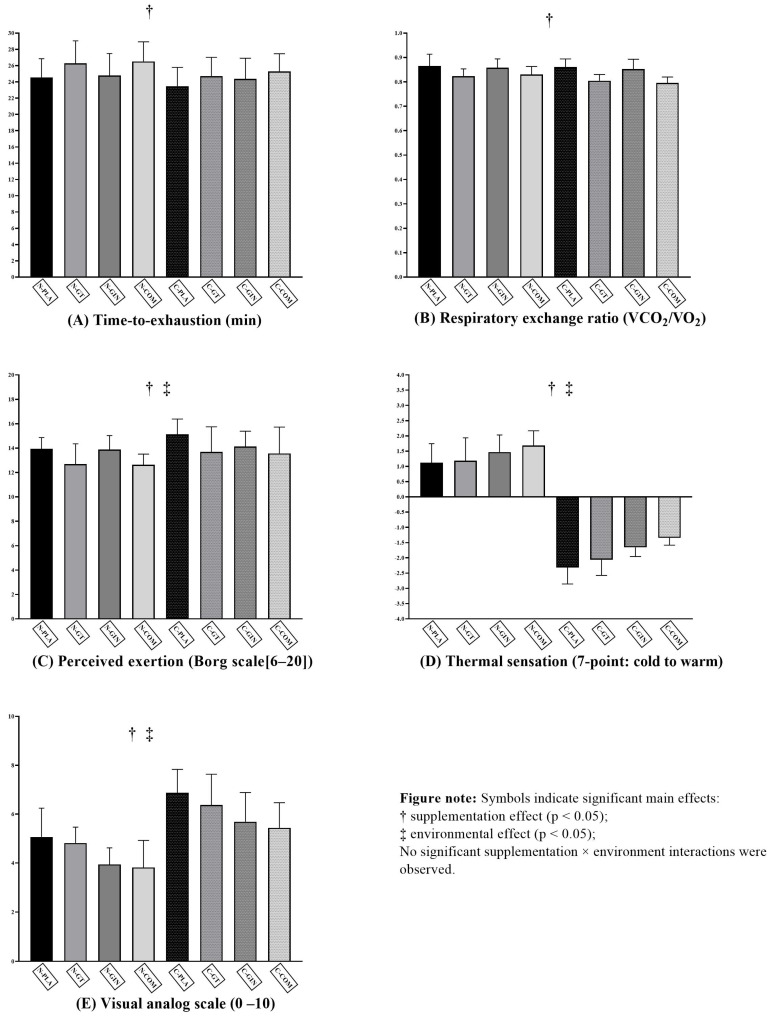
Effects of green tea (GT), ginger (Ging), combined supplementation (Comb), and placebo (PLA) under normothermic (Normo) and cold (Cold) conditions on (**A**) time-to-exhaustion, (**B**) respiratory exchange ratio, (**C**) rating of perceived exertion, (**D**) thermal sensation, and (**E**) muscle soreness assessed by visual analog scale. Values are presented as mean ± SD. Abbreviations: GT = Green Tea; Ging = Ginger; Comb = Combined; PLA = Placebo; Normo = Normothermic; Cold = Cold.

Descriptive and inferential statistics for all outcome variables are presented in Table 1, and a graphical summary of the primary and secondary outcomes is provided in Figure 1. Data for TTE were normally distributed, and sphericity was confirmed. A two-way repeated-measures ANOVA revealed a significant main effect of supplementation on TTE, indicating that supplementation influenced endurance performance. No significant main effect of the environmental condition was found. The interaction between supplementation and environmental condition was not significant. Post hoc pairwise comparisons identified significant differences (Table 1). In the normothermic environment (21–24 °C), green tea supplementation significantly increased TTE compared to the placebo (Hedges’ g = 1.28, *large effect*), as did the combined supplement (Hedges’ g = 1.43, *large effect*). Ginger supplementation showed no difference from the placebo. In the cold environment (5–7 °C), the combined supplement outperformed the placebo (Hedges’ g = 1.46, *large effect*), as did ginger (Hedges’ g = 1.14, *moderate effect*), and the combined supplement compared to the placebo (Hedges’ g = 2.40, *very large effect*). The combined supplement also outperformed ginger in the cold environment (Hedges’ g = 1.10, *moderate effect*). Cross-environment comparisons showed that cold–placebo significantly reduced TTE compared to normothermic–placebo (Hedges’ g = 1.76, large effect), normothermic–green tea (Hedges’ g = 2.16, *very large effect*), normothermic–ginger (Hedges’ g = 0.93, *moderate effect*), and normothermic–combined conditions (Hedges’ g = 2.31, *very large effect*). Normothermic–green tea outperformed cold–ginger (Hedges’ g = 1.24, *large effect*). Normothermic–combined outperformed cold–green tea (Hedges’ g = 1.04, *moderate effect*) and cold–ginger (Hedges’ g = 1.43, *large effect*). No other comparisons were significant.

Data for RER were normally distributed, and sphericity was confirmed. A two-way repeated measures ANOVA revealed a significant main effect of supplementation on RER, indicating that supplementation altered metabolic responses. No significant main effect of the environmental condition was found. The interaction between supplementation and environmental condition was not significant. Post hoc pairwise comparisons identified significant differences (Table 1). In the normothermic environment (21–24 °C), green tea supplementation significantly increased RER compared to the placebo (Hedges’ g = 1.06, *moderate effect*). In the cold environment (5–7 °C), both green tea (Hedges’ g = 1.12, *moderate effect*) and the combined supplement (Hedges’ g = 1.33, *large effect*) increased RER compared to the placebo. The combined supplement also outperformed ginger in the cold environment (Hedges’ g = 1.42, *large effect*), and cold–green tea outperformed cold–ginger (Hedges’ g = 1.28, *large effect*). Cross-environment comparisons showed that cold–green tea significantly increased RER compared to normothermic–placebo (Hedges’ g = 1.12, *moderate effect*), normothermic–ginger (Hedges’ g = 1.83, *large effect*), and normothermic–combined (Hedges’ g = 0.90, *moderate effect*). Cold–combined increased RER compared to normothermic–placebo (Hedges’ g = 1.33, *large effect*), normothermic–ginger (Hedges’ g = 2.01, *very large effect*), and normothermic–combined (Hedges’ g = 1.04, *moderate effect*). Normothermic–green tea outperformed cold–placebo (Hedges’ g = 0.99, *moderate effect*) and cold–combined (Hedges’ g = 0.97, moderate effect). Cold–placebo showed lower RER compared to cold–green tea (Hedges’ g = 1.99, *large effect*) and cold–combined (Hedges’ g = 2.01, *very large effect*). No other comparisons were significant.

Data for RPE were normally distributed, and sphericity was confirmed. A two-way repeated measures ANOVA revealed a significant main effect of supplementation on RPE, indicating that supplementation influenced perceived exertion. No significant main effect of environmental condition was found, and the interaction was not significant. Post hoc pairwise comparisons identified significant differences (Table 1). In the normothermic environment (21–24 °C), green tea supplementation significantly reduced RPE compared to the placebo (Hedges’ g = 1.11, *moderate effect*), as did the combined supplement (Hedges’ g = 1.77, *large effect*). The combined supplement also reduced RPE compared to ginger in the normothermic environment (Hedges’ g = 0.96, *moderate effect*). In the cold environment (5–7 °C), the combined supplement reduced RPE compared to the placebo (Hedges’ g = 0.93, *moderate effect*). Cross-environment comparisons showed that cold–placebo significantly increased RPE compared to normothermic–placebo (Hedges’ g = 1.35, *large effect*), normothermic–green tea (Hedges’ g = 1.70, *large effect*), normothermic–ginger (Hedges’ g = 0.92, *moderate effect*), and normothermic–combined conditions (Hedges’ g = 2.46, *very large effect*). Normothermic–combined also reduced RPE compared to cold–ginger (Hedges’ g = 1.23, *large effect*). No other comparisons were significant.

Data for TSS were normally distributed, and sphericity was confirmed. A two-way repeated-measures ANOVA revealed significant main effects of supplementation and environmental condition, indicating that both supplementation and environment influenced thermal sensation. The interaction between supplementation and environmental condition was not significant. Post hoc pairwise comparisons identified significant differences (Table 1). In the normothermic environment (21–24 °C), the combined supplement significantly reduced TSS compared to the placebo (Hedges’ g = 0.98, *moderate effect*) and green tea (Hedges’ g = 0.92, *moderate effect*). In the cold environment (5–7 °C), cold–green tea (Hedges’ g = 0.92, *moderate effect*), cold–ginger (Hedges’ g = 1.57, *large effect*), and cold–combined (Hedges’ g = 1.73, *large effect*) significantly reduced TSS compared to cold–placebo. Additionally, cold–green tea outperformed cold–ginger (Hedges’ g = 1.18, *moderate effect*), and cold–combined outperformed both cold–green tea (Hedges’ g = 1.32, *large effect*) and cold–ginger (Hedges’ g = 0.96, *moderate effect*). Cross-environment comparisons showed that all cold conditions significantly reduced TSS compared to all normothermic conditions. Specifically, cold–placebo reduced TSS compared to normothermic–placebo (Hedges’ g = 5.42, *very large effect*), normothermic–green tea (Hedges’ g = 4.86, *very large effect*), normothermic–ginger (Hedges’ g = 6.56, *very large effect*), and normothermic–combined (Hedges’ g = 7.86, *very large effect*). Cold–green tea reduced TSS compared to normothermic–placebo (Hedges’ g = 4.45, *very large effect*), normothermic–green tea (Hedges’ g = 4.22, *very large effect*), normothermic–ginger (Hedges’ g = 5.67, *very large effect*), and normothermic–combined (Hedges’ g = 6.89, *very large effect*). Cold–ginger reduced TSS compared to normothermic–placebo (Hedges’ g = 4.37, *very large effect*), normothermic–green tea (Hedges’ g = 3.73, *very large effect*), normothermic–ginger (Hedges’ g = 5.27, *very large effect*), and normothermic–combined (Hedges’ g = 6.26, *very large effect*). Cold–combined reduced TSS compared to normothermic–placebo (Hedges’ g = 3.50, *very large effect*), normothermic–green tea (Hedges’ g = 3.06, *very large effect*), normothermic–ginger (Hedges’ g = 4.43, *very large effect*), and normothermic–combined (Hedges’ g = 4.87, *very large effect*). No other comparisons were significant.

Data for VAS were normally distributed, and sphericity was confirmed. A two-way repeated-measures ANOVA revealed significant main effects of supplementation and environmental condition, indicating that both supplementation and environment influenced muscle soreness for 24 h post-exercise. The interaction between supplementation and environmental condition was not significant. Post hoc pairwise comparisons identified significant differences (Table 1). In the cold environment (5–7 °C), cold–green tea (Hedges’ g = 0.92, *moderate effect*), cold–ginger (Hedges’ g = 1.35, *large effect*), and cold–combined (Hedges’ g = 2.66, *very large effect*) significantly reduced VAS scores compared to cold–placebo. Cold–combine also reduced VAS scores compared to cold–green tea (Hedges’ g = 1.15, *moderate effect*). Cross-environment comparisons showed that cold–placebo significantly increased VAS scores compared to normothermic–placebo (Hedges’ g = 1.10, *moderate effect*), normothermic–green tea (Hedges’ g = 1.52, *large effect*), normothermic–ginger (Hedges’ g = 2.26, *very large effect*), and normothermic–combine (Hedges’ g = 1.85, *large effect*). Cold–green tea increased VAS scores compared to normothermic–green tea (Hedges’ g = 0.96, *moderate effect*), normothermic–ginger (Hedges’ g = 1.50, *large effect*), and normothermic–combined (Hedges’ g = 1.33, *moderate effect*). Cold–ginger increased VAS scores compared to normothermic–ginger (Hedges’ g = 1.16, *moderate effect*), normothermic–combined (Hedges’ g = 1.02, *moderate effect*), and cold–combined (Hedges’ g = 0.24, small effect, non-significant). Cold–combined increased VAS scores compared to normothermic–ginger (Hedges’ g = 1.18, *moderate effect*) and normothermic–combine (Hedges’ g = 1.03, *moderate effect*). No other comparisons were significant.

## 4. Discussion

The present study demonstrated that green tea extract and ginger combined supplementation-enhanced endurance performance, metabolic responses, and thermal perception in both normothermic and cold environments. The findings revealed that green tea and combined supplementation increased TTE, reduced RER to promote fat oxidation, and decreased RPE, with these effects being particularly pronounced in cold conditions. Cold exposure, as an independent factor, imposes distinct physiological demands; for instance, in the placebo condition, cold significantly elevated VAS compared to normothermic conditions, worsened TSS, and increased RPE. This reflects the disadvantages of cold-induced thermogenic stress and vasoconstriction, which elevate metabolic load, alongside advantages such as enhanced fat oxidation that improve energy efficiency [41,42]. The combination of green tea and ginger mitigated these disadvantages—evidenced by TSS’ improvement and reduced VAS—while amplifying the benefits, through a greater reduction in RER. This supportive combined effect likely comes from the optimization of thermogenic and ergogenic mechanisms mediated by caffeine, catechins, and gingerols, suggesting that appropriate supplementation can attenuate physiological decrements due to environmental stressors [15,16,22,43].

The ergogenic effects of green tea observed in normothermic conditions are derived from the complementary physiological actions of its primary constituents, namely catechins (notably epigallocatechin gallate, EGCG) and caffeine [14,17]. Specifically, catechins can activate AMP-activated protein kinase (AMPK), a key regulator of cellular energy homeostasis, which in chronic supplementation studies has been associated with enhanced lipolysis and mitochondrial biogenesis [44]. While such long-term adaptations are unlikely to arise from the acute, single-dose protocol employed in the present study, acute catechin intake may still transiently increase fatty acid oxidation and reduce reliance on carbohydrate stores during submaximal exercise, aligning with the observed reduction in respiratory exchange ratio [18,43,44]. Caffeine, meanwhile, acutely stimulates the central nervous system by antagonizing adenosine receptors, elevating dopamine and norepinephrine levels, and thereby reducing perceived exertion while prolonging TTE [45]. These acute mechanisms most plausibly explain the improved endurance performance recorded under green tea supplementation and are consistent with prior evidence supporting its lipolytic and stimulant properties [9,10,43]. Additionally, catechins enhance insulin sensitivity by modulating glucose uptake and insulin signaling pathways, optimizing energy substrate availability during exercise [10,46]. Their antioxidant properties mitigate further oxidative stress, delaying muscle fatigue and supporting sustained performance [10,47]. While caffeine may stimulate thermogenesis via brown adipose tissue activation, this effect appears less pronounced in normothermic conditions compared to colder environments, suggesting that green tea’s ergogenic benefits in this context rely predominantly on metabolic and neurological enhancements [45,48]. Collectively, these physiological processes support the study’s findings, highlighting the green tea’s capacity to optimize endurance and metabolic efficiency under normothermic environmental conditions.

Ginger resulted in improvements in TSS and VAS, particularly in cold conditions, primarily attributed to the physiological actions of its bioactive compounds, notably gingerols and shogaols, indicating a supportive role in thermal comfort and recovery rather than a direct ergogenic effect on endurance performance [23,49]. Gingerols are known for their thermogenic properties that stimulate transient receptor potential vanilloid 1 (TRPV1) channels, expressed in sensory neurons and vascular tissues [22,23]. Activation of TRPV1 enhances peripheral blood flow and induces mild heat production, counteracting the vasoconstriction and thermal discomfort induced by cold exposure [50]. In addition, gingerols may also interact with transient receptor potential melastatin 8 (TRPM8) channels, which are activated by menthol to evoke cooling sensations. Evidence suggests that gingerols can dampen TRPM8 activity, thereby attenuating cold perception and contributing to improved thermal comfort. This dual mechanism aligns with the observed TSS improvements after ginger supplementation, and is consistent with broader evidence on TRP channel modulation in environmental stress [51]. This mechanism aligns with TSS’ improvement after ginger supplementation, reflecting an enhanced perception of warmth. Additionally, gingerols upregulate peroxisome proliferator-activated receptor gamma coactivator 1-alpha (PGC-1α), a key regulator of mitochondrial biogenesis and thermogenesis, potentially amplifying heat generation through non-shivering thermogenesis in brown adipose tissue [52]. However, this thermogenic effect appears insufficient to significantly alter TTE or RER, as ginger alone did not induce different results compared to the placebo. Ginger’s anti-inflammatory properties, mediated by the inhibition of pro-inflammatory cytokines (e.g., TNF-α and IL-6) and cyclooxygenase-2 (COX-2) pathways, further contribute to its role in reducing VAS [53,54]. This attenuation of exercise-induced inflammation and muscle damage supports the study’s finding of decreased muscle soreness, consistent with prior evidence [50,55]. While ginger’s physiological actions enhance thermal comfort and recovery, its limited direct influence on energy metabolism explains the lack of standalone ergogenic effects, suggesting that its benefits are most pronounced when combined with other bioactive agents like green tea catechins.

The combined supplementation of green tea and ginger demonstrates a supportive combined effect, particularly in cold conditions, where it outperforms individual treatments in enhancing TTE, reducing RER, and improving TSS. This synergy arises from the complementary physiological actions of catechins, caffeine, and gingerols [15,54]. Green tea’s catechin content boosts lipolysis and mitochondrial fatty acid oxidation through AMPK activation, while caffeine amplifies this metabolic change induced by increasing catecholamine release (e.g., epinephrine), further mobilizing fat stores [56]. In cold environments, these effects are potentiated because non-shivering thermogenesis naturally elevates fat oxidation to maintain core temperature, which is evidenced by the significant RER reduction with the combined treatment [57]. Gingerols enhance this response by activating TRPV1 channels and upregulating PGC-1α, which collectively augment thermogenesis and peripheral circulation, mitigating cold-induced vasoconstriction [23,52]. This thermogenic synergy directly supports the TSS’ improvement, reflecting a heightened perception of warmth that complements green tea’s metabolic contributions [14,16,43]. The combined increase in TTE likely results from this optimized energy metabolism—where fat becomes the predominant fuel—coupled with caffeine’s ergogenic stimulation of the central nervous system, which sustains effort despite cold stress [45,48]. Furthermore, ginger’s anti-inflammatory properties may reduce subclinical muscle damage induced by cold, indirectly supporting endurance capacity [22,55]. This interplay aligns with the study’s observation of higher outcomes in the combined group, suggesting that the integration of thermogenic and metabolic adaptations offers a robust physiological advantage in challenging environmental conditions [58].

Previous research has established the individual effects of green tea and ginger on exercise performance [14,16,47,59], yet, our study uniquely highlights their synergistic potential, especially in cold environments. Studies on green tea, such as those systematically reviewed by Gholami et al. [43], demonstrated a modest enhancement of fat oxidation and endurance performance, with meta-analyses indicating small but consistent effects on body weight and fat reduction when combined with exercise. Similarly, the results from previous studies about the potential effect of green tea’s catechins in increasing fat oxidation during moderate-intensity exercise support those from our study highlighted by the reduced RER in both normothermic and cold conditions [10,44,46]. However, these studies primarily focused on weight loss or metabolic outcomes in warm environments, whereas our findings extend this to endurance and metabolic efficiency under cold stress, where the effect is amplified. Regarding ginger, Wilson [11,15] found that approximately 1–2 g/day modestly reduced muscle soreness post-exercise, particularly after eccentric or prolonged running, support the VAS reduction. Yet, these studies reported negligible ergogenic effects on performance metrics like TTE, mirroring ginger’s lack of potential impact in our trial. Sheikhhossein et al. [59] further supported the ginger’s role in mitigating oxidative stress, which may underpin its soreness relief, though this was not directly measured here. Unlike prior work, our study reveals that combining green tea and ginger not only enhances fat oxidation and endurance but also significantly improves thermal sensation in cold conditions—an outcome rarely explored previously—suggesting a novel application for athletes facing environmental challenges.

### Strengths and Limitations

This study presents several notable strengths that enhance its scientific rigor and relevance. The randomized, double-blind crossover design minimized bias and inter-individual variability, providing robust internal validity for assessing the effects of green tea and ginger supplementation across normothermic and cold conditions. The use of standardized environmental controls and precise physiological measurements, such as gas analysis for RER and validated scales for TSS and VAS, ensured reliable and reproducible data. Additionally, the inclusion of both individual and combined supplementation arms offered a comprehensive evaluation of their independent and supportive combined effects, a rare approach in prior research.

However, fundamental limitations must be acknowledged. The sample size of 16 male participants, while adequately powered for the primary outcomes, restricts the generalizability of findings to larger or more diverse populations. The study included only young, recreationally active men with VO_2_ max values corresponding to the average aerobic capacity for this demographic. Therefore, results cannot be extrapolated to women, older adults, or competitive athletes, whose hormonal, metabolic, and training characteristics may lead to different responses. The acute nature of the supplementation protocol (single dose with a short washout period) limits insights into chronic effects, which could differ given the potential for cumulative adaptations in fat oxidation or thermogenesis. Furthermore, the study did not assess molecular markers, such as oxidative stress or inflammatory cytokines, which could elucidate the underlying mechanisms of the observed benefits, particularly for VAS and TSS improvements.

A further limitation concerns the potential training effect associated with the experimental design. Participants completed nine separate exercise sessions, including multiple time-to-exhaustion trials, which may have represented a period of intensified training exposure. Although washout periods and the randomized crossover design reduced order and carryover effects, repeated exposure to TTE testing could still have induced training adaptations that influenced performance outcomes. As VO_2_ max was not reassessed post-intervention, this potential confounding factor cannot be confirmed or ruled out.

These constraints highlight the need for cautious interpretation and further investigation into broader applicability and long-term outcomes. Future research should address these limitations by including larger and more diverse populations (e.g., females), as well as chronic supplementation protocols to evaluate long-term adaptations. Moreover, incorporating biochemical markers of oxidative stress and inflammation could provide mechanistic insights into the observed effects.

## 5. Conclusions

The present study demonstrates that green tea extract and ginger supplementation, individually and in combination, enhanced endurance performance, metabolic efficiency, and thermal perception in recreationally active males under normothermic and cold conditions. Green tea alone increased time to exhaustion and reduced respiratory exchange ratio in normothermic conditions, indicating improved fat oxidation and endurance capacity. In cold conditions, the combination of green tea and ginger enhanced time to exhaustion, lowered the respiratory exchange ratio, improved thermal sensation, and reduced muscle soreness. These effects are most pronounced in cold environments, where the combined supplementation mitigated the heightened physiological demands of cold exposure while amplifying metabolic benefits. To our knowledge, this is the first randomized, double-blind crossover trial to systematically evaluate the supportive combined effects of green tea and ginger across different environmental conditions. This original contribution extends current evidence by demonstrating that nutritional strategies can not only support endurance under normothermic conditions but also attenuate cold-induced decrements in performance and comfort. The findings highlighted the potential of this supplementation strategy to optimize exercise performance and comfort, particularly under environmental stress. However, the benefits observed are specific to the acute supplementation protocol and male participants tested, underscoring the need for broader investigations to confirm these effects across diverse populations and longer durations. From a practical perspective, the doses employed in this study are within the range of commercially available supplements and generally regarded as safe for healthy adults. Nevertheless, individual tolerance and potential interactions, particularly related to caffeine intake, should be considered in applied settings. Furthermore, the current results reflect acute supplementation, and extrapolation to long-term use requires caution. Future studies should examine chronic supplementation protocols to evaluate safety, tolerability, and sustained efficacy.

## Figures and Tables

**Table 1:** Mean ± SD Values and Statistical Analysis Across Supplementation Conditions and Temperatures.

Variable	Placebo	Green Tea	Ginger	Combined	Statistics (F, *p*, ηp^2^)
TTE (Normothermic)	24.5 ± 2.3	26.3 ± 2.8 ^a^	24.8 ± 2.7	26.5 ± 2.4 ^a^	Supplementation effect: F = 27.274; *p* < 0.001; ηp^2^ = 0.476 Environmental condition effect: F = 1.741; *p* = 0.197; ηp^2^ = 0.055 Interaction effect: F = 2.316; *p* = 0.081; ηp^2^ = 0.072
TTE (s, Cold)	23.5 ± 2.3 ^abd^	24.7 ± 2.3 ^d^	24.4 ± 2.5 ^bd^	25.3 ± 2.2 ^aeg^	
RER (Normothermic)	0.86 ± 0.05	0.82 ± 0.03 ^a^	0.86 ± 0.04	0.83 ± 0.03	Supplementation effect: F = 29.976; *p* < 0.001; ηp^2^ = 0.500 Environmental condition effect: F = 3.142; *p* = 0.086; ηp^2^ = 0.095 Interaction effect: F = 2.200; *p* = 0.093; ηp^2^ = 0.068
RER (Cold)	0.86 ± 0.03 ^a^	0.80 ± 0.03 ^abcde^	0.85 ± 0.04 ^f^	0.80 ± 0.02 ^abcdeg^	
RPE (Normothermic)	13.94 ± 0.93	12.69 ± 1.66 ^a^	13.88 ± 1.15	12.62 ± 0.89 ^ac^	Supplementation effect: F = 13.572; *p* < 0.001; ηp^2^ = 0.311 Environmental condition effect: F = 4.094; *p* = 0.052; ηp^2^ = 0.120 Interaction effect: F = 1.212; *p* = 0.310; ηp^2^ = 0.039
RPE (Cold)	15.12 ± 1.26 ^abcd^	13.69 ± 2.06	14.12 ± 1.26 ^d^	13.56 ± 2.16 ^e^	
TSS (Normothermic)	1.12 ± 0.62	1.19 ± 0.75	1.47 ± 0.56	1.69 ± 0.48 ^ab^	Supplementation effect: F = 39.729; *p* < 0.001; ηp^2^ = 0.570 Environmental condition effect: F = 406.125; *p* < 0.001; ηp^2^ = 0.931 Interaction effect: F = 2.588; *p* = 0.058; ηp^2^ = 0.079
TSS (Cold)	−2.31 ± 0.54 ^abcd^	−2.06 ± 0.51 ^abcde^	−1.66 ± 0.30 ^abcdef^	−1.34 ± 0.24 ^abcdefg^	
VAS (Normothermic)	5.06 ± 1.18	4.81 ± 0.66	3.94 ± 0.68	3.81 ± 1.11	Supplementation effect: F = 24.463; *p* < 0.001; ηp^2^ = 0.449 Environmental condition effect: F = 34.225; *p* < 0.001; ηp^2^ = 0.533 Interaction effect: F = 0.196; *p* = 0.899; ηp^2^ = 0.007
VAS (Cold)	6.88 ± 0.96 ^abcd^	6.38 ± 1.26 ^bcde^	5.09 ± 1.20 ^cde^	5.44 ± 1.03 ^cdef^	

Values are expressed as mean ± SD. TTE = Time-to-Exhaustion (min). RER = Respiratory Exchange Ratio, calculated as VCO_2_/VO_2_. RPE = Rating of Perceived Exertion, assessed post-exercise using the Borg 6–20 scale. TSS = Thermal Sensation Scale, rated from −3 (very cold) to +3 (very warm). VAS = Visual Analog Scale of Delayed Onset Muscle Soreness, 0 (no pain) to 10 (extreme pain). Different lowercase letters (a–g) indicate significant differences (*p* < 0.05) between conditions based on post hoc analysis (Bonferroni correction). For example, “a” denotes a significant difference from Placebo (Normothermic), “b” from GT (Normothermic), “c” from Ging (Normothermic), “d” from Comb (Normothermic), “e” from Placebo (Cold), “f” from GT (Cold), “g” from Ging (Cold). Conditions sharing the same letter are not significantly different. GT = Green Tea, Ging = Ginger, Comb = Combined.

## Data Availability

The data presented in this study are available on request from the corresponding author due to institutional data-sharing policies.

## Abbreviations

The following abbreviations are used in this manuscript:

TTE	Time-to-exhaustion
RER	Respiratory exchange ratio
RPE	Perceived exertion
TSS	Thermal sensation
VAS	Visual analog scale
VO_2_	Oxygen consumption
ANOVA	Analysis of variance
BMI	Body mass index
VO_2_ max	Maximum oxygen consumption
CO_2_	Carbon dioxide
SD	Standard deviation
EGCG	Epigallocatechin gallate
AMPK	AMP-activated protein kinase
TRPV1	Transient receptor potential vanilloid 1
PGC-1α	Proliferator-activated receptor gamma coactivator 1-alpha
COX-2	Cyclooxygenase-2

## References

[1] Riera F., Bellenoue S., Fischer S., Méric H. (2021). Impact of a cold environment on the performance of professional cyclists: A pilot study. Life.

[2] Wallace P.J., Hartley G.L., Nowlan J.G., Ljubanovich J., Sieh N., Taber M.J., Gagnon D.D., Cheung S.S. (2024). Endurance capacity impairment in cold air ranging from skin cooling to mild hypothermia. J. Appl. Physiol..

[3] Ulupinar S., Özbay S., Gençoğlu C., Altinkaynak K., Şebin E., Oymak B. (2021). Exercise in the cold causes greater irisin release but may not be enough for adropin. J. Physiol. Investig..

[4] Ozbay S., Ulupınar S., Şebin E., Altınkaynak K. (2020). Acute and chronic effects of aerobic exercise on serum irisin, adropin, and cholesterol levels in the winter season: Indoor training versus outdoor training. J. Physiol. Investig..

[5] Sawka M.N., Young A.J. (2012). Physiologic systems and their responses to conditions of heat and cold. ACSM’s Advanced Exercise Physiology.

[6] Gatterer H., Dünnwald T., Turner R., Csapo R., Schobersberger W., Burtscher M., Faulhaber M., Kennedy M.D. (2021). Practicing sport in cold environments: Practical recommendations to improve sport performance and reduce negative health outcomes. Int. J. Environ. Res. Public Health.

[7] González-Gross M., Quesada-González C., Rueda J., Sillero-Quintana M., Issaly N., Díaz A.E., Gesteiro E., Escobar-Toledo D., Torres-Peralta R., Roller M. (2021). Analysis of effectiveness of a supplement combining Harpagophytum procumbens, Zingiber officinale and Bixa orellana in healthy recreational runners with self-reported knee pain: A pilot, randomized, triple-blind, placebo-controlled trial. Int. J. Environ. Res. Public Health.

[8] Huang J., Tagawa T., Ma S., Suzuki K. (2022). Black ginger (*Kaempferia parviflora*) extract enhances endurance capacity by improving energy metabolism and substrate utilization in mice. Nutrients.

[9] Zalakiyan P., Naghibi M. (2019). The Effect of Eight Weeks of Interval Aerobic Training with Green Tea and Ginger Consumption on Lipid Profiles of Overweight Women. Rep. Health Care.

[10] Nobari H., Saedmocheshi S., Chung L.H., Suzuki K., Maynar-Mariño M., Pérez-Gómez J. (2021). An overview on how exercise with green tea consumption can prevent the production of reactive oxygen species and improve sports performance. Int. J. Environ. Res. Public Health.

[11] Wilson P.B. (2020). A randomized double-blind trial of ginger root for reducing muscle soreness and improving physical performance recovery among experienced recreational distance runners. J. Diet. Suppl..

[12] Heck A.M., DeWitt B.A., Lukes A.L. (2000). Potential interactions between alternative therapies and warfarin. Am. J. Health-Syst. Pharm..

[13] Kondori B.J., Ghaleh H.E.G., Hosseini S.M. (2021). Effect of green tea extract on exercise-induced inflammatory markers. J. Mil. Med..

[14] Mashhadi M.R., Hosseini S.R.A. (2023). The interaction effect of green tea consumption and exercise training on fat oxidation, body composition and blood lipids in humans: A review of the literature. Sport Sci. Health.

[15] Wilson P.B. (2015). Ginger (*Zingiber officinale*) as an analgesic and ergogenic aid in sport: A systemic review. J. Strength Cond. Res..

[16] Hodgson A.B., Randell R.K., Jeukendrup A.E. (2013). The effect of green tea extract on fat oxidation at rest and during exercise: Evidence of efficacy and proposed mechanisms. Adv. Nutr..

[17] Mazyed E.A., Helal D.A., Elkhoudary M.M., Elhameed A.G.A., Yasser M. (2021). Formulation and optimization of nanospanlastics for improving the bioavailability of green tea epigallocatechin gallate. Pharmaceuticals.

[18] Tritsch N., Steger M.C., Segatz V., Blumenthal P., Rigling M., Schwarz S., Zhang Y., Franke H., Lachenmeier D.W. (2022). Risk assessment of caffeine and epigallocatechin gallate in coffee leaf tea. Foods.

[19] Unno K., Ikka T., Yamashita H., Kameoka Y., Nakamura Y. (2025). Stress-Relieving Effects of Japanese Green Tea: Evaluation Using the Molar Ratio of Caffeine and Epigallocatechin Gallate to Theanine and Arginine as an Indicator. Foods.

[20] Mashhadi N.S., Ghiasvand R., Askari G., Feizi A., Hariri M., Darvishi L., Barani A., Taghiyar M., Shiranian A., Hajishafiee M. (2013). Influence of ginger and cinnamon intake on inflammation and muscle soreness endued by exercise in Iranian female athletes. Int. J. Prev. Med..

[21] Standing J.F. (2017). Understanding and applying pharmacometric modelling and simulation in clinical practice and research. Br. J. Clin. Pharmacol..

[22] Samota M.K., Rawat M., Kaur M., Garg D. (2024). Gingerol: Extraction methods, health implications, bioavailability and signaling pathways. Sustain. Food Technol..

[23] Deng B., Jiang X.-L., Xu Y.-C., Chen S., Cai M., Deng S.-H., Ding W.-J., Xu H.-L., Zhang S.-W., Tan Z.-B. (2022). 10-Gingerol, a natural AMPK agonist, suppresses neointimal hyperplasia and inhibits vascular smooth muscle cell proliferation. Food Funct..

[24] McKay A.K.A., Stellingwerff T., Smith E.S., Martin D.T., Mujika I., Goosey-Tolfrey V.L., Sheppard J., Burke L.M. (2021). Defining training and performance caliber: A participant classification framework. Int. J. Sports Physiol. Perform..

[25] ACSM (2013). ACSM’s Guidelines for Exercise Testing and Prescription.

[26] Huggett D.L., Connelly D.M., Overend T.J. (2005). Maximal aerobic capacity testing of older adults: A critical review. J. Gerontol. Ser. A Biol. Sci. Med. Sci..

[27] Faulkner J., Parfitt G., Eston R. (2007). Prediction of maximal oxygen uptake from the ratings of perceived exertion and heart rate during a perceptually-regulated sub-maximal exercise test in active and sedentary participants. Eur. J. Appl. Physiol..

[28] Borg G. (1970). Perceived exertion as an indicator of somatic stress. Scand. J. Rehabil. Med..

[29] Gagge A.P., Stolwijk J.A.J., Hardy J.D. (1967). Comfort and thermal sensations and associated physiological responses at various ambient temperatures. Environ. Res..

[30] Huskisson E.C. (1974). Measurement of pain. Lancet.

[31] Wang Z., Cao B., Ji W., Zhu Y. (2020). Study on clothing insulation distribution between half-bodies and its effects on thermal comfort in cold environments. Energy Build..

[32] Ioannou L.G., Tsoutsoubi L., Gkiata P., Brown H.A., Periard J.D., Mekjavic I.B., Kenny G.P., Nybo L., Flouris A.D. (2024). Effect of sportswear on performance and physiological heat strain during prolonged running in moderately hot conditions. Scand. J. Med. Sci. Sports.

[33] Mifflin M.D., St Jeor S.T., Hill L.A., Scott B.J., Daugherty S.A., Koh Y.O. (1990). A new predictive equation for resting energy expenditure in healthy individuals. Am. J. Clin. Nutr..

[34] Sacks F.M., Bray G.A., Carey V.J., Smith S.R., Ryan D.H., Anton S.D., McManus K., Champagne C.M., Bishop L.M., Laranjo N. (2009). Comparison of weight-loss diets with different compositions of fat, protein, and carbohydrates. N. Engl. J. Med..

[35] Espinosa-Salas S., Gonzalez-Arias M. (2023). Nutrition: Micronutrient intake, imbalances, and interventions. StatPearls [Internet].

[36] Cohen J. (2013). Statistical Power Analysis for the Behavioral Sciences.

[37] Calónico S., Galiani S. (2025). Beyond Bonferroni: Hierarchical Multiple Testing in Empirical Research.

[38] Hopkins W.G., Marshall S.W., Batterham A.M., Hanin J. (2009). Progressive statistics for studies in sports medicine and exercise science. Med. Sci. Sports Exerc..

[39] Brydges C.R. (2019). Effect size guidelines, sample size calculations, and statistical power in gerontology. Innov. Aging.

[40] Ulupınar S., İnce İ. (2021). Effect size and alternative statistical approaches in sports sciences. Spormetre J. Phys. Educ. Sport Sci..

[41] van der Lans A.A., Hoeks J., Brans B., Vijgen G.H., Visser M.G., Vosselman M.J., Hansen J., Jörgensen J.A., Wu J., Mottaghy F.M. (2013). Cold acclimation recruits human brown fat and increases nonshivering thermogenesis. J. Clin. Investig..

[42] Xu J., Cui L., Wang J., Zheng S., Zhang H., Ke S., Cao X., Shi Y., Li J., Zen K. (2023). Cold-activated brown fat-derived extracellular vesicle-miR-378a-3p stimulates hepatic gluconeogenesis in male mice. Nat. Commun..

[43] Gholami F., Antonio J., Iranpour M., Curtis J., Pereira F. (2024). Does green tea catechin enhance weight-loss effect of exercise training in overweight and obese individuals? A systematic review and meta-analysis of randomized trials. J. Int. Soc. Sports Nutr..

[44] Mika M., Wikiera A., Antończyk A., Grabacka M. (2021). The impact of catechins included in high fat diet on AMP-dependent protein kinase in apoE knock-out mice. Int. J. Food Sci. Nutr..

[45] Sharma V.K., Sharma A., Verma K.K., Gaur P.K., Kaushik R., Abdali B. (2023). A comprehensive review on pharmacological potentials of caffeine. J. Appl. Pharm. Sci. Res..

[46] Yanagimoto A., Matsui Y., Yamaguchi T., Hibi M., Kobayashi S., Osaki N. (2022). Effects of ingesting both catechins and chlorogenic acids on glucose, incretin, and insulin sensitivity in healthy men: A randomized, double-blinded, placebo-controlled crossover trial. Nutrients.

[47] Matsuzaki R., Matsuoka T., Nakanishi K., Tani A., Kakimoto S., Kato Y., Kawatani T., Nakagawa S., Baba Y., Kobayashi M. (2025). Effects of green tea catechins and exercise on age-related muscle atrophy and satellite cell functions in a mouse model of sarcopenia. Exp. Gerontol..

[48] Alsabri S.G., Mari W.O., Younes S., Elsadawi M.A., Oroszi T.L. (2018). Kinetic and dynamic description of caffeine. J. Caffeine Adenosine Res..

[49] Gao Y., Lu Y., Zhang N., Udenigwe C.C., Zhang Y., Fu Y. (2024). Preparation, pungency and bioactivity of gingerols from ginger (*Zingiber officinale* Roscoe): A review. Crit. Rev. Food Sci. Nutr..

[50] Liu X., Meng X., Su X., Ren K., Ning C., Qi X., Zhang S. (2022). The mechanism of ginger and its processed products in the treatment of estradiol valerate coupled with oxytocin-induced dysmenorrhea in mice via regulating the TRP ion channel-mediated ERK 1/2/NF-κB signaling pathway. Food Funct..

[51] Barwood M.J., Gibson O.R., Gillis D.J., Jeffries O., Morris N.B., Pearce J., Ross M.L., Stevens C., Rinaldi K., Kounalakis S.N. (2020). Menthol as an Ergogenic aid for the Tokyo 2021 Olympic games: An Expert-Led consensus statement using the modified Delphi method. Sports Med..

[52] Peng Z., Zeng Y., Zeng X., Tan Q., He Q., Wang S., Wang J. (2024). 6-Gingerol improves lipid metabolism disorders in skeletal muscle by regulating AdipoR1/AMPK signaling pathway. Biomed. Pharmacother..

[53] Yahyazadeh R., Rahimi V.B., Yahyazadeh A., Mohajeri S.A., Askari V.R. (2021). Promising effects of gingerol against toxins: A review article. Biofactors.

[54] Yücel Ç., Karatoprak G.Ş., Açıkara Ö.B., Akkol E.K., Barak T.H., Sobarzo-Sánchez E., Aschner M., Shirooie S. (2022). Immunomodulatory and anti-inflammatory therapeutic potential of gingerols and their nanoformulations. Front. Pharmacol..

[55] Black C.D., Herring M.P., Hurley D.J., O’Connor P.J. (2010). Ginger (*Zingiber officinale*) reduces muscle pain caused by eccentric exercise. J. Pain.

[56] Rocha A., Bolin A.P., Cardoso C.A.L., Otton R. (2016). Green tea extract activates AMPK and ameliorates white adipose tissue metabolic dysfunction induced by obesity. Eur. J. Nutr..

[57] Nishimura T., Motoi M., Egashira Y., Choi D., Aoyagi K., Watanuki S. (2015). Seasonal variation of non-shivering thermogenesis (NST) during mild cold exposure. J. Physiol. Anthropol..

[58] Hursel R., Westerterp-Plantenga M.S. (2010). Thermogenic ingredients and body weight regulation. Int. J. Obes..

[59] Sheikhhossein F., Borazjani M., Jafari A., Vataniyan E., Gholami F., Amini M.R. (2021). Effects of ginger supplementation on biomarkers of oxidative stress: A systematic review and meta-analysis of randomized controlled trials. Clin. Nutr. ESPEN.

